# Multiscale Entropy of Resting-State Functional Magnetic Resonance Imaging Differentiates Progressive Supranuclear Palsy and Multiple System Atrophy

**DOI:** 10.3390/life11121411

**Published:** 2021-12-16

**Authors:** Katsuhiko Kadota, Keiichi Onoda, Satoshi Abe, Chizuko Hamada, Shingo Mitaki, Hiroaki Oguro, Atsushi Nagai, Hajime Kitagaki, Shuhei Yamaguchi

**Affiliations:** 1 Department of Neurology, Faculty of Medicine, Shimane University, Izumo 693-8501, Japan; sabe@med.shimane-u.ac.jp (S.A.); okuzihc@med.shimane-u.ac.jp (C.H.); Shingomi@med.shimane-u.ac.jp (S.M.); oguro@med.shimane-u.ac.jp (H.O.); anagai@med.shimane-u.ac.jp (A.N.); yamagu3n@med.shimane-u.ac.jp (S.Y.); 2 Department of Psychology, Otemon Gakuin University, Osaka 567-8502, Japan; onodak1@gmail.com; 3 Department of Radiology, Faculty of Medicine, Shimane University, Izumo 693-8501, Japan; kitagaki@med.shimane-u.ac.jp

**Keywords:** progressive supranuclear palsy, multisystem, resting-state functional MRI, multiscale entropy, frontal executive function, rs-fMRI, neural network, connectivity

## Abstract

Distinguishing progressive supranuclear palsy (PSP) from multiple system atrophy (MSA) in the early clinical stages is challenging; few sensitive and specific biomarkers are available for their differential diagnosis. Resting-state functional magnetic resonance imaging (rs-fMRI) is used to study the fluctuations in blood oxygen level-dependent (BOLD) signals at rest, which provides evidence for aberrant brain functional networks in neurodegenerative diseases. We aimed to examine whether rs-fMRI data could differentiate between PSP and MSA via a multiscale entropy (MSE) analysis of BOLD signals, which estimates the complexity of temporal fluctuations in brain activity. We recruited 14 and 18 patients with PSP and MSA, respectively, who underwent neuropsychological tests and rs-fMRI. PSP patients demonstrated greater cognitive function impairments, particularly in the frontal executive function. The bilateral prefrontal cortex revealed lower entropy BOLD signal values in multiple time scales for PSP, compared to the values observed in MSA patients; however, the functional connectivity of the representative brain networks was comparable between the diseases. The reduced complexity of BOLD signals in the prefrontal cortex was associated with frontal dysfunction. Thus, an MSE analysis of rs-fMRI could differentiate between PSP and MSA, and the reduced complexity of BOLD signals could be associated with cognitive impairment.

## 1. Introduction

Progressive supranuclear palsy (PSP) and multiple system atrophy (MSA) are neurodegenerative diseases with diverse brain pathologies: PSP is characterized by tau filaments with four repeats, globular neurofibrillary changes, and glial fiber changes in the astrocytes and oligodendrocytes; conversely, MSA is characterized by fibrillar inclusions of α-synuclein (termed glial cytoplasmic inclusions) in the oligodendrocytes [1,2]. However, both of their gross pathological features display atrophy in common regions, such as the brain stem, cerebellum, basal ganglia, and frontal lobe, which represent the structural basis for clinical magnetic resonance imaging (MRI) examination [3]. Patients with early-stage PSP and MSA often exhibit similar clinical features, including parkinsonism (bradykinesia, rigidity, and postural instability) and cognitive impairment without specific MRI abnormalities. Cognitive and behavioral changes are also common and usually represent frontal executive dysfunction, including apathy, impulsivity, inattention, personality changes, and reduced processing speed. An autopsy study revealed that a group of patients clinically diagnosed with MSA included few PSP cases (15/134, 11.2%) [4]. Therefore, a definitive clinical diagnosis between PSP and MSA is occasionally difficult [5,6].

Recently, the resting-state functional MRI (rs-fMRI) technique using spontaneous neuronal activity has revealed functional brain networks [7]. Regions that are functionally related or co-activated during a cognitive task display temporally correlated activities at rest. Spontaneous neuronal activities are estimated by slow fluctuations in the blood oxygen level-dependent (BOLD) signals, and functional neural networks are represented by spatial maps of the correlations of the aforementioned signal fluctuations between anatomically separate brain regions. These highly correlated brain regions are functionally connected, and the strength of their connections is represented by the correlation values between specific regions. Functional connectivity is reportedly associated with the severity of dementia and aging-related illnesses, and cognitive decline [8]. For example, the default mode network (DMN), which includes a core network comprising the medial prefrontal cortex and the posterior cingulate cortex, is involved in the pathophysiology of Alzheimer’s disease [9], thereby suggesting that rs-fMRI data could contribute to the understanding and diagnosis of neurodegenerative diseases.

Researchers have demonstrated the changes in functional connectivity occurring in PSP and MSA using rs-fMRI techniques. The extensive disruption of multiple brain functional networks in PSP has been revealed in various cortical and subcortical regions, including the dorsal midbrain tegmentum, thalamus, caudate nucleus, putamen, and pallidum [9,10,11,12,13]. The affected network in MSA is widely distributed in brain regions, including the primary sensorimotor cortex, anterior and posterior cingulate cortex, lateral prefrontal cortex, dorsomedial prefrontal cortex, basal ganglia, and cerebellum [14,15,16]. Thus, a substantial number of large-scale networks are involved in the pathophysiology of both PSP and MSA. However, no reports have directly compared the changes in the brain functional networks between PSP and MSA. In this study, we aimed to perform a direct comparison of the functional connectivity using the conventional region of interest (ROI)-to-ROI analysis in PSP and MSA to elucidate the differences in the functional connectivity patterns.

Furthermore, we aimed to apply a different type of analysis to the rs-fMRI dataset, namely, the Multiscale Entropy (MSE) analysis. The MSE has recently been used in the field of biomedical signal processing, such as the analysis of electrocardiograms (ECG), electroencephalograms (EEG), and MRI imaging. In particular, it is useful for studying neural network mechanisms. The MSE analysis can reveal the dynamic complexity of the time series of the signals in biological systems over various time scales, especially those occurring in BOLD signals [17]. Therefore, we applied MSE to investigate the dynamic complexity of the time series of BOLD signals over multiple time scales. It was developed to distinguish between random noise and complex signals. The entropy of the former signal decreases with an increase in the time scale, whereas the entropy of the latter signal is maintained because of self-similarity across time scales. MSE is used as a validation metric to quantify the complexity of rs-fMRI signals. Several studies have utilized MSE to quantify the complexity of BOLD signals in the brains of the elderly, reporting its usefulness [18,19]. Grieder et al. [20] investigated functional connectivity within the DMN and MSE decline in patients with mild Alzheimer’s disease. Their measurements significantly correlated with those of cognitive impairment. We hypothesized that there may be some difference in the MSE between PSP and MSA associated with the changes in cognitive function, despite similar patterns of functional connectivity. We also intended to examine the relationship between the MSE metrics and cognitive functions assessed by neuropsychological tests in these patient groups.

## 2. Materials and Methods

### 2.1. Patients

We recruited 14 patients with PSP and 18 with MSA who had been referred to the Department of Neurology at the Shimane Medical University Hospital. All patients had one or more symptoms of parkinsonism, cerebellar ataxia, postural retention disorders, dementia, and visited Shimane Medical University Hospital for diagnosis and scrutiny. They were evaluated by neurologists specializing in neurodegenerative diseases and were clinically diagnosed with PSP or MSA. Diagnoses of probable PSP were based on the criteria of the National Institute of Neurological Disorders and Stroke and the Society for PSP. Diagnoses of probable MSA were based on the criteria of the University of Michigan’s Second Consensus Conference on MSA [21,22]. The severity of PSP and MSA was assessed using a modified Rankin Scale (mRS) based on the unified Parkinson’s Disease Rating Scale for PSP and the International Cooperative Ataxia Rating Scale for MSA. The aforementioned scales were not directly compared because we used the mRS to assess the effect of the illness severity or burden on the activities of daily living. Table 1 summarizes the patient demographics. All patients provided informed consent, and this study was approved by the Shimane University Medical Ethics Committee (Protocol code: 657).

### 2.2. Neuropsychological Assessment

All patients were assessed using neuropsychological test batteries, including the Mini-Mental State Examination (MMSE) [23], frontal assessment battery (FAB) [24], self-rating depression scale (SDS) [25], and Apathy Scale (AS) [26,27]. These tests were conducted by a trained clinical psychologist within 1 month of the MRI examination. The cut-off values for these tests are 23/24 for MMSE, 10 for FAB, 40 for SDS, and 16 for AS [25,27,28,29]. Two patients (one per group) could not undergo FAB owing to their clinical status.

### 2.3. Image Acquisition

We used a General Electric 3.0T scanner to acquire the brain MRI data. First, all patients underwent rs-fMRI examinations for a total of 5 min, and were instructed to stay awake, relax, and remain calm with their eyes closed during the examination. We used T2-weighted, gradient echo, spiral pulse sequence (repetition time = 2000 ms, echo time = 35 ms, flip angle = 90°, scan order = interleaved, matrix size = 64 × 64, field of view (FOV) = 220 × 220 mm^2^, isotropic spatial resolution = 3.4 × 3. 4 mm, slice = 20/29, slice thickness = 3 mm, and gap = 0.5/1.5 mm) to measure 20 axial slices parallel to the plane connecting the anterior commissure. Following the functional scans, we recorded T1-weighted images of the entire brain (192 sagittal slices, repetition time = 7.1 ms, echo time = 2.1 ms, inversion time = 700 ms, spacing between slices = 0.8 mm, flip angle = 12°, matrix size = 512 × 512, FOV = 220 × 220 mm^2^, and isotropic spatial resolution = 0.43 mm). Moreover, the ordinal clinical MRIs were obtained and submitted to multiple neuroradiologists for evaluations of the brain structural changes, such as atrophy, silent brain infarct, cerebral microbleeds, periventricular hyperintensity, and deep and subcortical white matter hyperintensity.

### 2.4. Processing for Functional Imaging

We performed a statistical parametric mapping for preprocessing. The first five functional images of each patient were discarded to permit magnetic field stabilization and patient adaptation to the scanning environment. The remaining 145 functional images were readjusted to eliminate head movement artifacts and corrected for the differences in the image acquisition time between slices. The functional image was normalized to the standard space defined by the template T1-weighted image and resliced to a voxel size of 3 × 3 × 3 mm^3^. Spatial preprocessing was followed by temporal preprocessing using a functional connectivity toolbox (Conn: fMRI functional connectivity toolbox). We first performed temporal smoothing by filtering with a 0.01–0.08 Hz passband. Subsequently, the time series of head motion, white matter signals, and cerebrospinal fluid signals were regressed from each voxel.

### 2.5. ROI-to-ROI Analysis

Functional connectivity analysis based on the ROI is the most basic approach for rs-fMRI data. We conducted an ROI-to-ROI analysis to examine the differences in functional connectivity between PSP and MSA. To define the brain nodes, we used an automated anatomical labeling (AAL) atlas to divide the entire brain, except the cerebellum, into 90 regions. The average time courses of the voxels in each region were extracted, and a network was constructed. We calculated the Pearson correlation coefficient for each edge for all possible pairs extracted from the functionally connected regions. To simplify the calculation, we performed a Fisher r-to-z transformation to increase the normality of the correlation matrix, wherein the z-score was considered the functional connectivity between the ROIs. We subsequently compared the connectivity matrix using the network-based statistic (NBS) toolbox. In the NBS analysis, we performed a two-sample t-test with the age, sex, and disease duration as covariates. The threshold was set to three, and the number of permutations was 1000.

### 2.6. MSE

**Figure 1:**
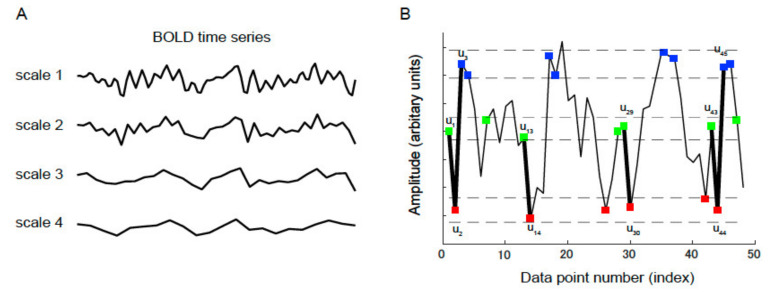
(**A**) Applying the coarse-graining process to a time series. For a certain time series, multiple coarse-grained time series are constructed by averaging the data in non-overlapping windows of progressively increasing length. (**B**) To illustrate the procedure for calculating the sample entropy, a time series (u) is displayed. The length of the pattern (m) is set to 2, and the criterion of similarity (r) is specified as 0.3 of the standard deviation of u. The dotted horizontal lines surrounding u_1_, u_2_, and u_3_ indicate u_1_ ± r, u_2_ ± r, and u_3_ ± r, respectively. Two data values are comparable, and if the absolute difference is <r, they are indistinguishable. The green, red, and blue points coincide with u_1_, u_2_, and u_3_, respectively. The m-component green-red template (u_1_, u_2_) and (m + 1)-component green-red-blue (u_1_, u_2_, u_3_) template sequences are considered. The segment comprises three green-red sequences matching the template; however, only one green-red-blue sequence matches the template. In this case, sequences three and one match the two- and three-component templates, respectively. These calculations are repeated for the next two- and three-component template sequences. The numbers of sequences that match each of the aforementioned components are added to the previous values. This procedure is repeated for all possible templates to determine the ratio of the total number of matches between the two- and three-component templates. The sample entropy is the natural logarithm of this ratio.

Sample entropy proposed by Richman and Moorman [30] is an analytical method devised as “constant invariant statistics” for measuring the regularity of the time series behaving in a complex manner (Figure 1). The sample entropy could be evaluated from random variation and the time series date. Sample entropy is defined by the negative natural logarithm of the conditional probability that a time series data of pattern length m, having repeated itself within a tolerance of r (similarity factor), will also repeat itself for m + 1 points, without allowing self-matches [30]. MSE evaluates the complexity of longer time-scale fluctuations by filtering out high-frequency fluctuations through a procedure that averages t-consecutive points to create a new time series of length N/t, where t denotes the time scale.

The Complexity Toolbox (http://loft-lab.org/index-5.html, accessed on 13 December 2021) was used to calculate the MSE of the rs-fMRI data. In the MSE calculation, we set the pattern length m, the distance threshold r, and the time scale l. For short data sets (time series length −100), we used a sample entropy of r ≥ 0.3 (which agrees well with the theoretical value when m = 2) [30]. While previous fMRI studies set the sample entropy to m = 1 or 2, and used r = 0.30–0.45 [31,32,33], we computed the MSE for each BOLD time series in this study based on m = 2 and r = 0.3 across scales from 1 to 4.

We performed analyses of covariance with the age, sex, and measurement period as covariates to compare the entropy maps of each scale between the PSP and MSA groups. The statistical criteria were set to a false discovery rate-corrected *p* < 0.05 at the cluster level and uncorrected *p* < 0.001 at the voxel level. We subsequently extracted the individual data in spheres of 6 mm radius centered on the peak voxel of a significant cluster, which was used for the visualization and subsequent analyses. All statistical analyses were performed using IBM SPSS Statistics version 26 (IBM, Armonk, NY, USA).

## 3. Results

Table 1 summarizes the demographic information, neuropsychological test scores, and pathological findings on MRI. There were no significant differences in age and sex between the PSP and MSA groups. The mean disease duration was slightly longer for patients with MSA than were those for patients with PSP; however, the difference was not significant. Moreover, the groups did not reveal differences in the mRS scores. We incorporated the age, sex, and disease duration as covariates while analyzing the rs-fMRI data. The MMSE and FAB-measured cognitive functions were lower in the PSP than those in the MSA group; however, only FAB exhibited statistical significance (MMSE: *p* = 0.051, FAB: *p* = 0.004). The SDS and AS-assessed affective functions did not reveal any significant differences between the groups. We also estimated the incidence of silent brain infarction, cerebral microbleeds, periventricular hyperintensity, and deep and subcortical white matter hyperintensity, and did not observe significant differences in these pathological MRI findings between the groups.

**Figure 2:**
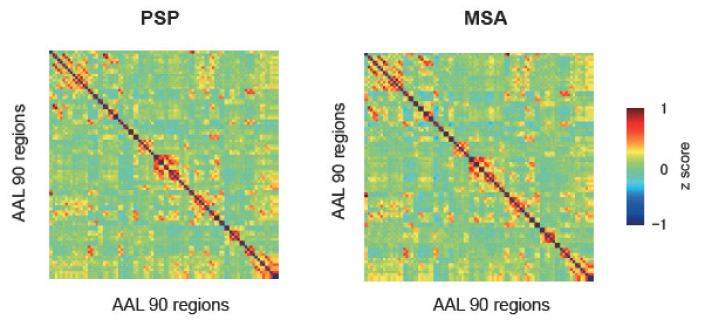
Functional connectivity between 90 regions of the automated anatomical labelling (AAL) in progressive supranuclear palsy (PSP) and multiple system atrophy (MSA). Red and blue denote positive and negative connectivity, respectively.

We conducted ROI-to-ROI analysis, which is one of the basic analyses for rs-fMRI data, to assess the functional connectivity between the multiple brain regions. We compared the functional connectivity matrix of 90 regions between PSP and MSA based on the AAL atlas. Both groups demonstrated similar functional connectivity maps in the group-level analysis (Figure 2). There were no significant differences in the connectivity patterns between the groups in the network-based analysis.

**Figure 3:**
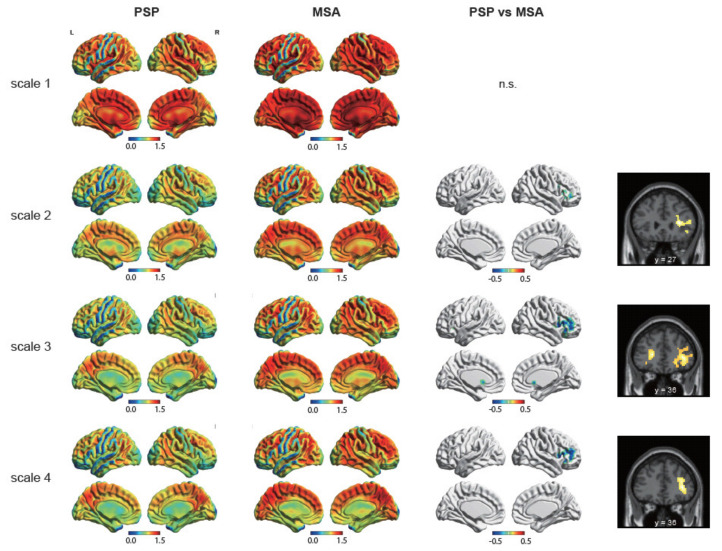
Columns 1 and 2 present the average maps of the sample entropy (lateral and medial views) for scales 1–4 for progressive supranuclear palsy (PSP; first column) and multiple system atrophy (MSA; second column). Columns 3 and 4 represent the group comparisons of sample entropy using entire-brain t-test (false discovery rate-corrected *p* < 0.05 at the cluster level and uncorrected *p* < 0.001 at the voxel level).

We subsequently compared the MSE between the groups. Figure 3 presents the lateral and medial views of the MSE for each scale. For all scales, the entropy of the frontal, temporoparietal junction, and medial regions were relatively higher than those of the other regions. A comparison of the entropy between the groups revealed a robust decrease in the entropy in the bilateral prefrontal cortex for PSP (Table 2). The lateral part of the right prefrontal cortex exhibited lower entropy across the scales (2–4). Conversely, the left prefrontal cortex revealed a lower entropy for PSP than that for MSA across scale 3.

**Figure 4:**
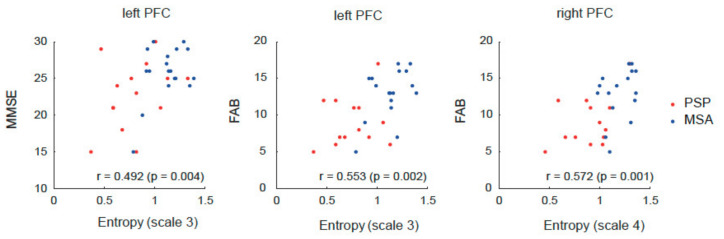
Correlations between multi scale entropy and neuropsychological scores. PFC, prefrontal cortex; MMSE, mini-mental examination; FAB, frontal assessment battery. Each point represents an individual patient with progressive supranuclear palsy (PSP, red) or multiple system atrophy (MSA, blue).

Furthermore, we performed correlation analyses between the MSE values and neuropsychological test scores as well as summarized the results in Table 3. We extracted the entropy values from a 6 mm sphere centered at the region with the peak value, and calculated the mean entropy values. Correlation analyses revealed an association between the entropies in the prefrontal cortex and cognitive function. In other words, the entropy of the left prefrontal cortex on scale 3 positively correlated with the MMSE (r = 0.49, *p* = 0.004) and FAB (r = 0.55, *p* = 0.002) scores. Moreover, the entropy of the right prefrontal cortex on scale 4 also revealed a significant positive correlation with the FAB (r = 0.57, *p* = 0.001) scores (Figure 4). However, neither SDS nor AS exhibited significant correlations with the entropies in the prefrontal cortex.

## 4. Discussion

In the clinical setting, it is occasionally difficult to distinguish PSP from MSA in the early stages owing to similar clinical features, such as extra-pyramidal symptoms. Furthermore, the patients exhibit similar cognitive impairment patterns; specifically, they commonly present with frontal executive dysfunction. However, we observed significantly greater impairment of frontal function in patients with PSP, compared to those with MSA. Notably, the difference in frontal executive functions alone is not a reliable index for the differential diagnosis of these diseases.

In this study, we compared the brain network metrics between PSP and MSA using rs-fMRI data. Despite extensive studies on network changes in each disease using healthy subjects as a comparison group, to our knowledge, this is the first study to elucidate the differences in the functional brain networks between PSP and MSA. Previous studies have demonstrated the involvement of a substantial number of large-scale common networks in the pathophysiology of PSP and MSA, such as the prefrontal cortex, basal ganglia, thalamus, midbrain, and cerebellum [9,10,11,12,13,14,15,16]. Consistent with these reports, the current study revealed similar changes in brain networks in the disease process of PSP and MSA.

Despite no clear difference in the brain network types affected by PSP and MSA with the ROI-to-ROI or network-based analyses of BOLD signals at rest, our study demonstrated a significant difference between the two diseases in the entropy analysis. Entropy analysis is a nonlinear signal processing technique that provides a measure to probe the complexity of signal dynamics, such as electroencephalography, magnetoencephalography, or fMRI. The entropy value reflects the randomness and predictability of a stochastic process. Thus, increased values are associated with greater randomness, and a lower value indicates a lower complexity of the signal or system [34]. Our results indicated that signal complexity was considerably reduced in the bilateral prefrontal cortex in PSP compared to that observed in MSA. In particular, the right lateral prefrontal cortex displayed a significant decrease in complexity in patients with PSP in multiple time window ranges. Furthermore, neuropsychological test scores, particularly the FAB score, positively correlated with the entropy value in the prefrontal cortex. The reduced complexity of BOLD signals in the prefrontal cortex in patients with PSP was associated with low FAB scores. Our results support the previously established significant relationship between cognitive function and the complexity of BOLD activity at rest.

Several studies involving entropy analyses have demonstrated an association between age-related decline in cognitive function and reduced complexity in multiple brain regions, including the subcortical regions. Moreover, cognitive decline has been correlated with reduced complexity in the subcortical regions, which may involve reduced information transfer between the cortical and subcortical regions in the form of reduced functional or anatomical connectivity [35]. However, our study revealed differences limited in the entropy value, but not in the connectivity pattern among the brain regions between the two diseases. This discrepancy does not coincide with the notion of a close relationship between signal complexity and functional connectivity in the rs-fMRI data analyses. The physiological nature of the complexity in spontaneous BOLD signals remains unclear, thus warranting an analysis of the complexity of spontaneous brain activity in relation to functional connectivity.

This study demonstrated a greater reduction in neuropsychological test scores in PSP compared to those in MSA; however, the difference was insignificant. By contrast, the entropy analysis of BOLD signals demonstrated a robust difference between the diseases in terms of complexity in the right prefrontal cortex. Therefore, the entropy value of BOLD signals could complement the clinical diagnosis of neurological disorders. It seems noteworthy to test the hypothesis that an entropy analysis could identify subtle and subclinical functional brain impairment, which is otherwise difficult to elucidate.

Several limitations should be considered when interpreting our findings. First, a pathological confirmation of PSP and MSA diagnosis was lacking. We selected patients with highly probable diagnoses based on criteria widely used in clinical neurology. Second, while PSP is currently classified into eight clinical subtypes, MSA is subclassified into MSA-P and MSA-C. Considering the relatively small sample size, we could not conduct a subgroup analysis for each patient group. An MSE analysis for rs-fMRI data could provide group-level evidence for PSP diagnosis; however, further studies are warranted to extend the applicability of this analysis to an individual-level diagnosis of PSP or MSA.

## 5. Conclusions

In summary, the MSE values of BOLD signals at rest in the prefrontal cortex could differentiate PSP from MSA. Patients with PSP exhibited reduced complexity of signals compared to those of patients with MSA, and this reduction was associated with greater impairment of frontal executive function. Although the relationship between the strength of functional connectivity and MSE values of rs-fMRI data warrants further study, MSE measurements are presumed to be highly accurate in detecting subtle changes in brain functional network activity.

## Figures and Tables

**Table 1:** Background characteristics of the patients.

	PSP	MSA	*p*-Value
N	14	18	
Age (years)	73.5 ± 6.2	69.3 ± 8.7	0.132
Sex (female/male)	9/5	10/8	0.618
Disease duration (years)	3.3 ± 1.7	5.1 ± 3.2	0.073
mRS	3.2 ± 0.8	3.5 ± 0.8	0.750
MMSE	22.8 ± 4.6	25.8 ± 3.7	0.051
FAB	9.1 ± 3.4	13.2 ± 3.6	0.004
SDS	45.1 ± 10.4	39.5 ± 11.4	0.161
AS	17.6 ± 6.7	14.8 ± 8.2	0.317
SBI (%)	14.3	11.1	0.425
CMB (%)	7.1	11.1	0.702
PVH (0/1/2, %)	12.5/42.9/28.6	33.3/38.9/27.8	0.956
DSWMH (0/1/2/3, %)	21.4/35.7/28.6/14.3	27.8/22.2/33.6/16.7	0.868
Head movement (mm)	0.77 ± 0.57	0.69 ± 0.58	0.715

Numerical data are shown as mean ± standard deviation. The t-test is used for the numerical data, including age, disease duration, neuropsychological scores, and head movement during resting-state fMRI. The χ^2^ test is used to assess the sex and brain indices. mRS, modified Rankin scale; MMSE, Mini-Mental State Examination; FAB, Frontal Assessment Battery; SDS, Self-rating Depression Scale; AS, Apathy Scale; SBI, silent brain infraction; CMB, cerebral microbleeds; PVH, periventricular hyperintensity; DSWMH, deep and subcortical white matter hyperintensity.

**Table 2:** Brain regions displaying decreased entropy in PSP compared to MSA.

	Scale 2			Scale 3			Scale 4		
	[x, y, z]	Size	p_FDR_	[x, y, z]	Size	p_FDR_	[x, y, z]	size	*p*_FDR_
Left PFC	-	-	-	[−26, 36, 9]	223	0.041	-	-	-
Right PFC	[33, 27, 9]	486	0.005	[39, 36, 3]	828	<0.001	[39, 36, 6]	364	<0.001

PSP, progressive supranuclear palsy; MSA, multiple system atrophy; PFC, prefrontal cortex; FDR, false discovery rate [x, y, z] indicates the Montreal Neurological Institute (MNI) coordinates.

**Table 3:** Correlation between the multiscale entropy and neuropsychological scores.

		MMSE	FAB	SDS	AS
Scale 2	PFC (R)	0.142	0.416	0.026	−0.095
Scale 3	PFC (L)	0.492 *	0.553 *	0.004	−0.084
	PFC (R)	0.181	0.393	−0.112	−0.066
Scale 4	PFC (R)	0.353	0.572 *	−0.078	−0.092

* false discovery rate (FDR)-corrected *p* < 0.05 (uncorrected *p* < 0.004). MMSE, Mini-Mental State Examination; FAB, Frontal Assessment Battery; SDS, Self-rating Depression Scale; AS, apathy scale; PFC, prefrontal cortex.

## Data Availability

The data presented in this study are available on request from the corresponding author. The data are not publicly available owing to privacy protection of the patients.
